# Atlas of Cellular Dynamics during Zebrafish Adult Kidney Regeneration

**DOI:** 10.1155/2015/547636

**Published:** 2015-05-18

**Authors:** Kristen K. McCampbell, Kristin N. Springer, Rebecca A. Wingert

**Affiliations:** Department of Biological Sciences and Center for Zebrafish Research, University of Notre Dame, Notre Dame, IN 46556, USA

## Abstract

The zebrafish is a useful animal model to study the signaling pathways that orchestrate kidney regeneration, as its renal nephrons are simple, yet they maintain the biological complexity inherent to that of higher vertebrate organisms including mammals. Recent studies have suggested that administration of the aminoglycoside antibiotic gentamicin in zebrafish mimics human acute kidney injury (AKI) through the induction of nephron damage, but the timing and details of critical phenotypic events associated with the regeneration process, particularly in existing nephrons, have not been characterized. Here, we mapped the temporal progression of cellular and molecular changes that occur during renal epithelial regeneration of the proximal tubule in the adult zebrafish using a platform of histological and expression analysis techniques. This work establishes the timing of renal cell death after gentamicin injury, identifies proliferative compartments within the kidney, and documents gene expression changes associated with the regenerative response of proliferating cells. These data provide an important descriptive atlas that documents the series of events that ensue after damage in the zebrafish kidney, thus availing a valuable resource for the scientific community that can facilitate the implementation of zebrafish research to delineate the mechanisms that control renal regeneration.

## 1. Introduction 

The vertebrate kidney is comprised of functional units known as nephrons, which are epithelial tubules that cleanse the bloodstream of metabolic waste through vascular filtration and subsequent urine production [1]. Vertebrates form up to three kidney structures that are comprised of nephrons during development, termed the pronephros, mesonephros, and metanephros [1, 2]. A conserved trait of nephrons among these kidney forms across diverse terrestrial and aquatic vertebrate species is that they display a fundamentally similar regional organization along their length, containing a renal corpuscle that serves to filter blood, proximal and distal tubule segments that are specialized to perform discrete tasks in solute reabsorption and secretion, and a collecting duct that transports urine out of the organ and modifies salt and water levels [3, 4].

Acute kidney injury (AKI) is a devastating and often lethal condition in which nephron epithelial cells are destroyed by damage from ischemia or toxin exposure, typically affecting proximal tubule segments [5]. While there is compelling evidence from work in various fish and mammalian models that vertebrate nephron epithelial tubule cells can be robustly regenerated after some forms of AKI damage [6, 7], there is still a poor understanding of the mechanisms that mediate this regeneration response, and there are ongoing controversies regarding the cell(s) of origin that enable kidney regeneration in different species [2, 8, 9].

**Figure 1:**
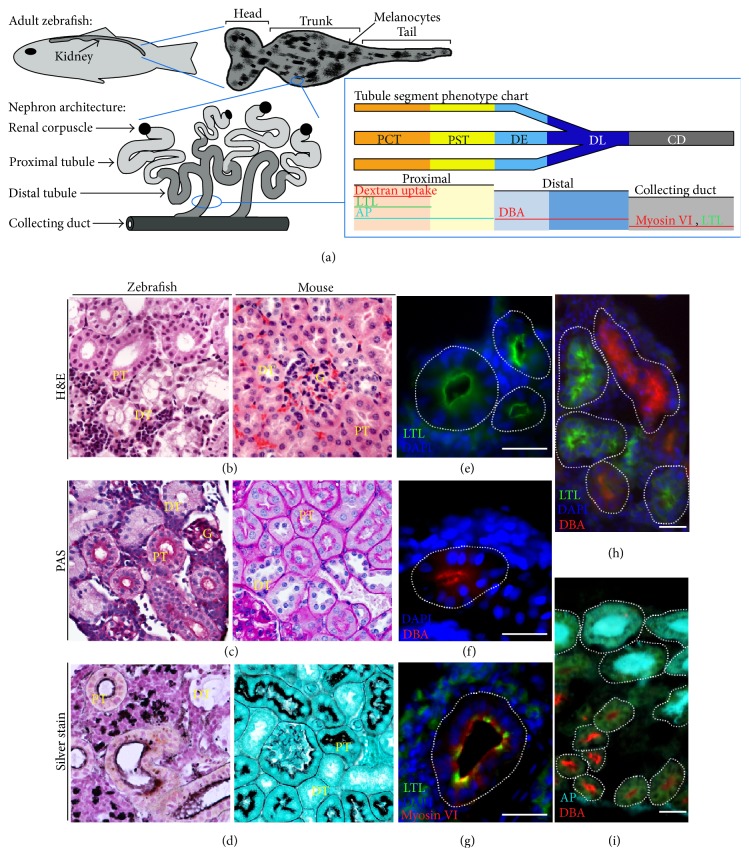
Anatomy of the zebrafish kidney and the identification of histological stains to distinguish renal structures. (a) The adult zebrafish kidney is comprised of arborized arrangements of nephrons that share common distal late tubule segments and drain into major collecting ducts (schematic adapted from [32]). ((b)–(d)) Histological staining in the zebrafish kidney revealed similarities to that of mammalian organ structure. (b) H&E and (c) PAS staining of wild-type zebrafish and mouse kidney tissue emphasized the brush border and elongated cells characteristic of the proximal tubule (PT, yellow label) compared to the pale pink hue of the distal tubule (DT, yellow label). Blood filters or glomeruli (G, yellow label) were as indicated. (d) Silver staining highlighted the brush border in a deep brown hue and additionally stained hyaline droplets located in the PT, while DT structures lacked the brown labeling. Zebrafish tissue sections 60x, mouse tissue sections 40x. ((e)–(i)) Fluorescent labeling of zebrafish tubules and collecting duct, with perimeters of each respective structure outlined in white dots, and nuclei ((e)–(h)) labeled with DAPI. Scale bars, 25 *μ*m. (e) LTL (green) stained the PT, while (f) DBA (red) stained the DT, and these tubule populations are mutually exclusive (h). (g) LTL also stained the collecting ducts, which were distinguished by a myosin VI antibody. (i) In transgenic* Tg:enpep:egfp* zebrafish, PT structures labeled with ELF-97 to detect alkaline phosphatase were mutually exclusive to DBA-stained distal tubules, and tubules were identified by labeling with anti-eGFP (representative panel reprinted with permission from [32]).

The zebrafish,* Danio rerio*, has emerged as a genetically tractable vertebrate model to study renal biology and associated medical conditions such as AKI, both in the embryonic and in adult settings [10]. The zebrafish embryo kidney, which is functional pronephros, consists of a pair of segmented nephrons that share a blood filter and each contains two proximal and two distal tubule segments [11, 12]. This structure forms by 1 day post fertilization (dpf) and blood filtration commences at approximately 2 dpf [13, 14], thus proffering a rapid and anatomically simple system for research on nephron patterning [15–18], identification of essential genes [11–13, 19], tubulogenesis [20, 21], and physiology and disease modeling [22–26]. In comparison, the adult zebrafish kidney, or mesonephros, is a single, relatively flat organ attached to the dorsal body wall that consists of characteristic bilaterally symmetric regions referred to as the head (or anterior), trunk (or medial), and tail (or posterior) (Figure 1(a)) [27]. The mesonephros begins to form between about 12 and 14 dpf, with the progressive addition of nephrons to the existing pronephric pair [28]. Over the lifespan of the zebrafish, the mesonephros continues to accumulate nephrons—a phenomenon linked to their continual adult growth (measured in tip to tail length of the animal) and the associated increasing excretory demands [28]. In the typical zebrafish adult at around 6 months of age, this mesonephric kidney is estimated to possess approximately 450 nephrons, with the most densely populated sites of nephrons in the head and trunk [28]. The adult nephrons have similar segments as found in the pronephros but are grouped in branched arrangements (Figure 1(a)) [29], which like other fishes do not show a regular orientation of nephrons as seen in the cortex and medulla of the mammalian metanephric kidney [6]. Zebrafish mesonephric nephrons commonly have shared distal tubule segments (Figure 1(a)) and drain into a pair of major collecting ducts that span the length of the organ [10, 29]. The stroma, consisting of the cells interspersed between mesonephric nephrons, is the site of adult hematopoiesis and also contains an intriguing populace of mesenchymal renal progenitors [10, 28, 29]. These renal progenitors provide a continual source of new nephrons that are produced in a process termed nephron neogenesis or neonephrogenesis, which occurs during the aforementioned processes of mesonephros development and when the mesonephros grows in response to naturally increasing biomass of the aging fish [10, 28, 29].

Overall, the complexity of the zebrafish mesonephric kidney provides a useful adult setting for renal biology studies and shows promise for identifying genetic components of the renal regeneration response that can complement research in traditional mammalian AKI models such as the mouse and rat [10]. To date, kidney regeneration paradigms in adult zebrafish have included nephrotoxin administration, specifically of the aminoglycoside antibiotic gentamicin to induce widespread nephron tubule damage [10, 28, 29], as well as the creation of several transgenic strains that were engineered to induce ablation of particular epithelial cell types in the nephron blood filter [30, 31]. Among the former, previous studies have demonstrated that two major events transpire following gentamicin-induced AKI in the adult zebrafish kidney organ: (1) neonephrogenesis, or the production of new nephrons due to the activation of the aforementioned stromal renal progenitors, which is detectable by histology based on the appearance of basophilic nephron units [10, 28, 29], and (2) partial functional restoration in existing nephrons around 4 days post injury (dpi), suggestive that the damaged nephron tubule epithelium recovers rather rapidly from chemical insult [29]. Despite these observations, the precise temporal sequence of molecular events that transpire within zebrafish nephrons during AKI, including cell death and proliferation, has received relatively little scrutiny. Some alterations in gene expression have been annotated in prior work, such as the transient abrogation of transcripts encoding* slc20a1a *[29], a sodium-dependent phosphate transporter that is normally localized to the proximal tubule epithelium, but further observations have been quite limited.

A significant impediment toward the pursuit of characterizing kidney regeneration aspects in zebrafish has been the paucity of histological and other molecular labeling methodologies tailored for use in this model organism. More recently, we have adapted a number of techniques to illuminate renal structures that can now be utilized [32]. For example, we demonstrated that the various proximal tubule segments could be distinguished by unique combinations of lectin staining, dextran uptake, and alkaline phosphatase (AP) reactivity (Figure 1(a)) [32]. Further, distal tubules are distinguished by labeling with a different lectin (Figure 1(a)) [32].

Here, to obtain a more detailed understanding of the regeneration events associated with zebrafish renal injury and identify cellular attributes enabling the demarcation of tubule segments, we performed extensive histological and immunofluorescence studies to annotate the sequence of tissue changes that result following gentamicin nephrotoxicity. For this work, we used and/or modified several traditional histology protocols for use with zebrafish renal tissues and also implemented a number of our recently developed nephron labeling methodologies [32]. With these tools, we now demonstrate for the first time that following gentamicin-induced AKI, nephron proximal tubule epithelial regeneration proceeds over approximately one week. We have now documented the detailed succession of cell death and proliferation in proximal tubules during this interval. Through additional structural and functional assays, we show that proximal tubules throughout the post-AKI zebrafish kidney regain absorptive capacity between 14 and 21 dpi. Further, we show that neonephrogenesis occurs in a partially overlapping time frame as nephron epithelial regeneration, beginning around 5 dpi and progressing over the subsequent two weeks, and show that regenerating populations in both existing and new nephrons express the renal transcription factor Pax2. Taken together, these descriptive and functional studies provide an essential foundation for future work aimed at elucidating the mechanisms that regulate kidney regeneration following AKI in the adult zebrafish.

## 2. Results 

**Figure 2:**
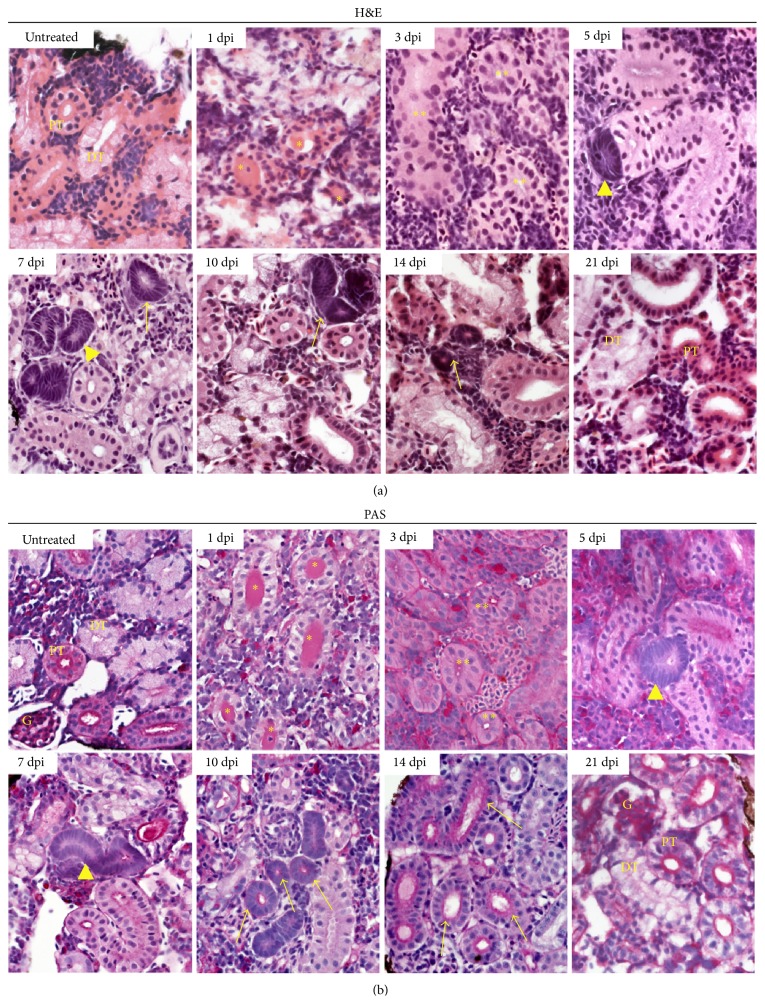
Histological staining reveals the process of regeneration after gentamicin-induced injury in adult zebrafish. (a) H&E and (b) PAS staining demonstrated extensive nephron damage and tubule destruction, followed by regenerative events that were completed by 21 dpi. The formation of basophilic cellular aggregates (darkest purple cellular staining) was indicative of new nephron formation. Both time courses were completed over a three-week period. 60x. DT: distal tubule; G: glomerulus; PT: proximal tubule. Yellow labels: asterisk (∗) indicates luminal debris; double asterisk (∗∗) indicates restoration of cells to tubules; arrowhead indicates neonephrogenic cluster; arrow indicates neonephron with visible lumen.

**Figure 3:**
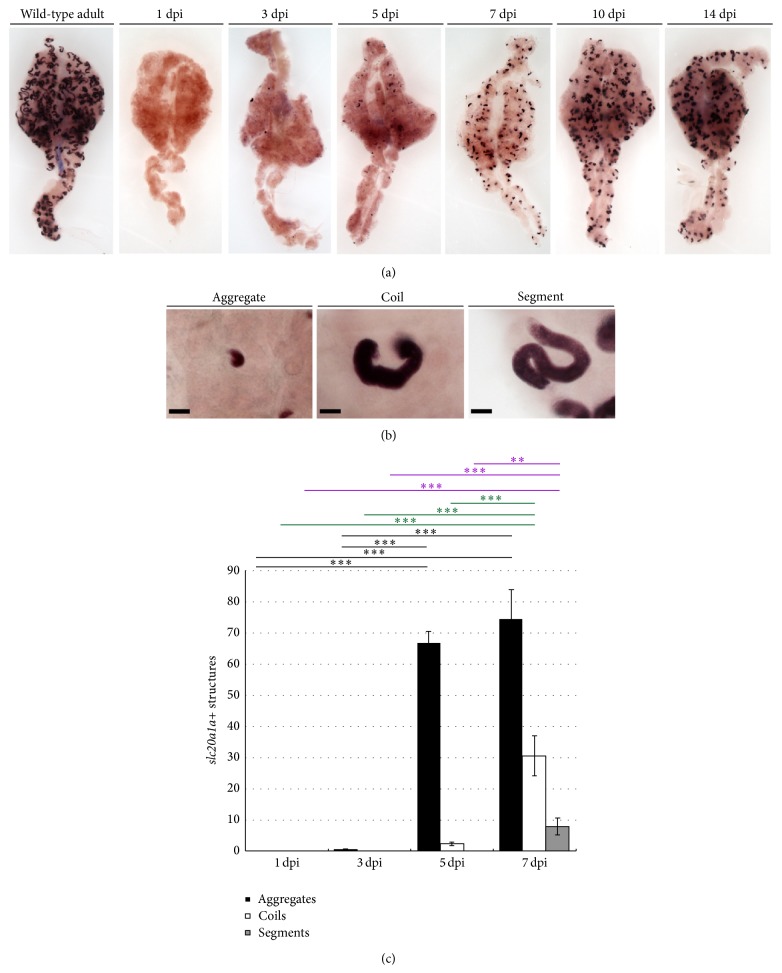
Dynamics of* slc20a1a* expression over the course of zebrafish adult kidney injury and regeneration. (a) Whole mount* in situ *hybridization of adult kidneys over a two-week injury time course revealed the initial absence of* slc20a1a* transcripts, followed by an increase in transcript levels and domains to near-normal levels by 14 dpi. Throughout the time course, new nephrons first appeared as small cellular aggregates that form coils and eventually elongated into PCT segments that are indistinguishable from preexisting, regenerated PCT tubules. 3x magnification. (b) Representative images of the progressive stages of nephron formation. Scale bar, 25 *μ*m. (c) Quantification shows that* slc20a1a* positive structures during regeneration. Labels: ∗∗ indicates *P* < 0.01; ∗∗∗ indicates *P* < 0.001; black indicates aggregate; green indicates coil; purple indicates segment.

**Figure 4:**
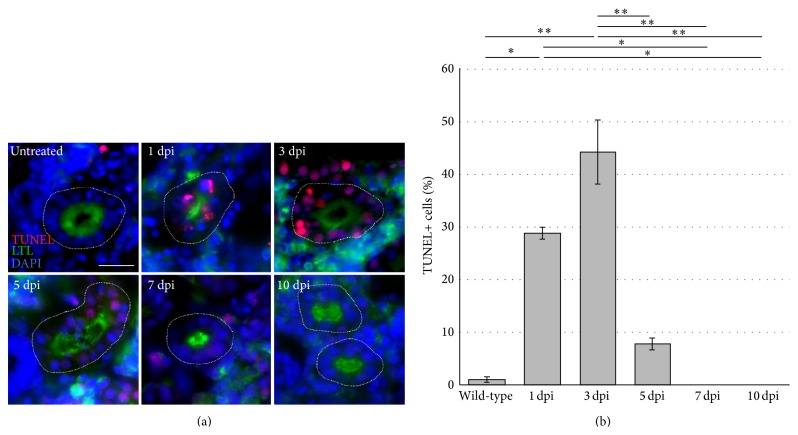
Detection of proximal tubule cell death after gentamicin injury. (a) Kidney tissue was assayed for TUNEL-positive cells in uninjured kidneys and at 1, 3, 5, 7, and 10 dpi. The peak of TUNEL reactivity in cells was identified within LTL-positive proximal tubules at 1, 3, and 5 days after kidney injury. By 7 and 10 days, the level of TUNEL-positive cells decreased, returning to basal levels previously established in untreated kidneys. Proximal tubules were labeled by LTL. (b) Quantification of TUNEL labeling in nephron proximal segments. Scale bar, 25 *μ*m. Labels: ∗ indicates *P* < 0.05; ∗∗ indicates *P* < 0.01.

**Figure 5:**
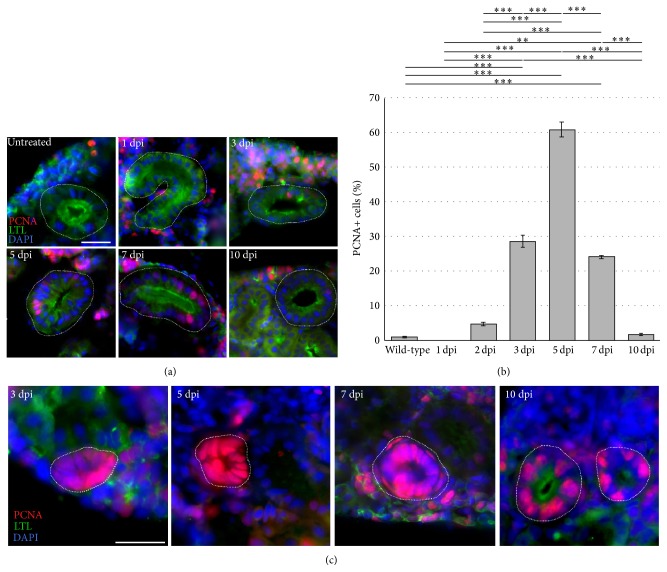
Cell proliferation in the regenerating proximal tubule epithelium and in neonephrogenic clusters. Kidney tissue was assayed for PCNA-positive nuclei (red), and proximal tubules were identified by LTL labeling (green). Nuclei were stained with DAPI (blue). (a) Most epithelial proliferation occurred at 3, 5, and 7 days after kidney injury. Scale bar, 25 *μ*m. (b) Quantification of PCNA expression in nephron proximal tubular segments. Labels: ∗∗ indicates *P* < 0.01; ∗∗∗ indicates *P* < 0.001. (c) Epithelial proliferation marked by PCNA (red) occurred at high levels in cellular aggregates at 3 and 5 dpi. At 7 dpi, aggregates that have formed lumens continued to express intense levels of PCNA. At 10 dpi, PCNA was still abundant in tubules that have become LTL-positive (green).

**Figure 6:**
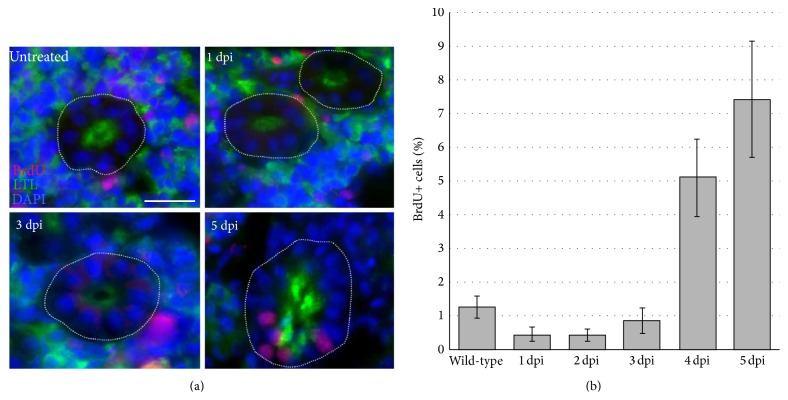
Regenerating proximal tubules show BrdU incorporation after gentamicin injury. (a) Kidney tissue was assayed for BrdU-positive nuclei (red), and proximal tubules were identified by LTL labeling (green). Nuclei were stained with DAPI (blue). (b) Quantification showed that BrdU incorporation occurs progressively at 3, 4, and 5 dpi.

**Figure 7:**
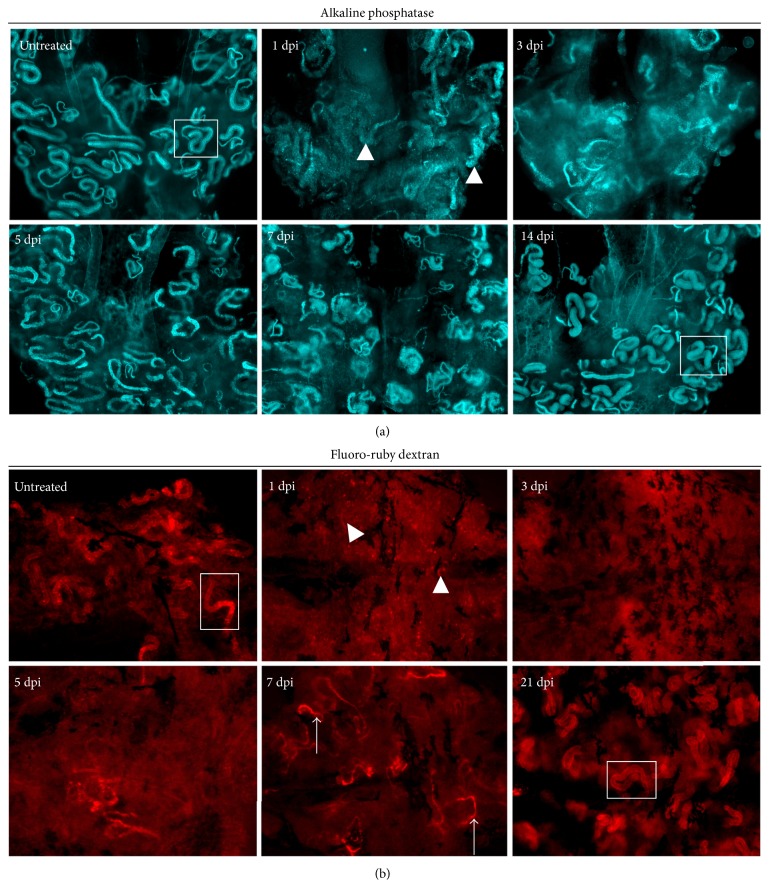
Changes in ELF-97 staining and proximal tubule functionality after gentamicin injury. Whole mount labeling of (a) alkaline phosphatase reactivity and (b) uptake of fluoro-ruby dextran in the zebrafish kidney during recovery from AKI. (a) Untreated kidneys contained discrete PCT-PST structures (white box) that were destroyed at 1 dpi (white arrowheads). Restoration of discrete PCT-PST structures was completed by 14 dpi. (b) Nephron reabsorption was visualized in injured kidneys, in which the PCT was demarcated by uptake of fluoro-ruby dextran, while at 1 dpi, such structures were absent (white arrowheads). Partial uptake was sporadically observed at 5–7 dpi but was not consistent throughout the kidney organ until 21 dpi when PCT segments were distinctly visualized (white box).

**Figure 8:**
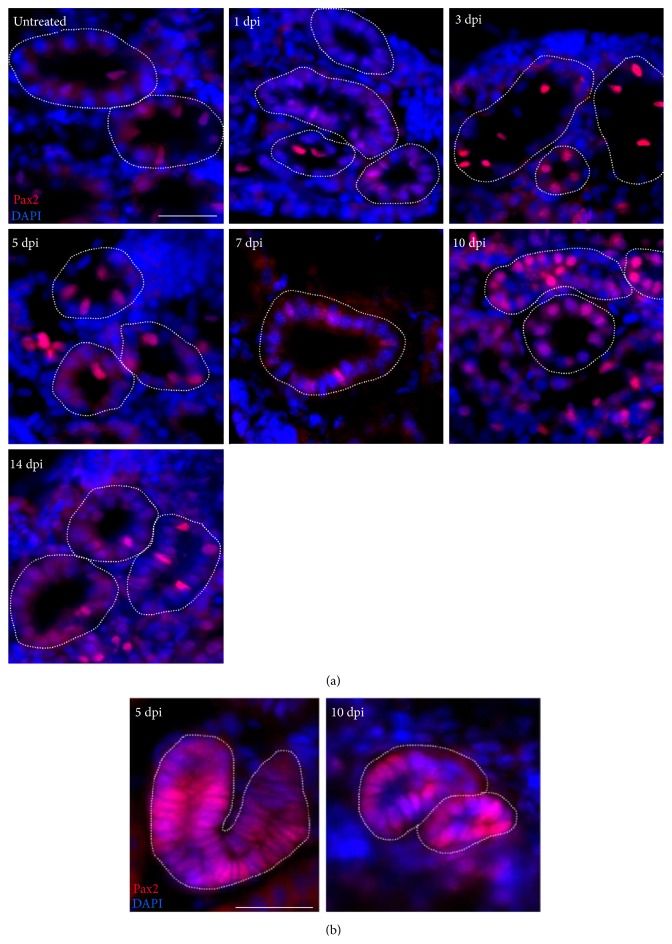
The Pax2 transcription factor marks cells in the regenerating proximal tubule and neonephrogenic clusters. (a) Expression of Pax2 in the regenerating kidney tubule. Pax2-positive nuclei (red) were identified in kidney tubule cryosections at baseline and over a two-week span after injury. Elevated Pax2 expression was detected at 5, 7, and 10 dpi. Nuclei were stained with DAPI (blue). Scale bar, 25 *μ*m. (b) Intense Pax2 immunoreactivity demarcated neonephrogenic units. High levels of Pax2 expression (red) were present in coil-like structures at 5 dpi. At 10 dpi, Pax2 continued to stain profound numbers of cells in the coil-like neonephron structures. Nuclei are stained with DAPI (blue). Scale bar, 25 *μ*m.

The aminoglycoside antibiotic gentamicin is an established nephrotoxin that has been used to model AKI by inducing renal tubule damage in the adult zebrafish [10, 28, 29], as well as other fish species, such as goldfish and medaka [6, 33]. In the latter studies, the histology of nephron phenotypes after gentamicin administration was documented, including assessment of cell proliferation by such measures as detection of proliferating cell nuclear antigen (PCNA) labeling and the incorporation of 5-bromo, 2′deoxyuridine (BrdU) [6, 33]. Previous studies in zebrafish have documented the appearance of gentamicin-damaged nephrons at 1 dpi and the incorporation of BrdU in immature nephrons [29], but the sequence of cellular alterations in gentamicin-damaged nephrons over time has not been determined. To examine and document such cellular changes, we first adapted histological methods that would enable characterization of renal structures (Figure 1) and then utilized these and other approaches to explore the spatial and temporal sequence of proximal tubule cell death and proliferation (Figures 2–8).

### 2.1. Histological Stains Distinguish Renal Structures

The adult zebrafish kidney is comprised of pinwheel-like arrangements of nephrons in which several nephrons connect to individual branched distal tubules that drain into the collecting ducts (Figure 1(a)) [10, 29, 32]. Hematoxylin and eosin (H&E) staining is a basic method that distinguishes the proximal tubule from the distal tubule based partly on the presence of a brush border: the proximal tubule possesses a brush border, whereas distal tubules do not [34]. The brush border, found on the luminal side of the proximal tubule epithelial cells, is lined with densely packed microvilli, forming a surface that greatly increases the surface area of the cells, facilitating their reabsorption functions. When paraffin sections of the kidney from healthy adult zebrafish were stained with H&E, the brush border was prominent as a thick pink stained band at the apical side of dark-pink stained proximal tubule cells, which showed characteristic elongated shapes, and formed a dilated lumen (Figure 1(b)). In comparison, the cells of the distal tubule had a narrow lumen and stained with a much lighter pink hue, allowing clear distinction of segment identity (Figure 1(b)). H&E staining in the healthy adult murine kidney revealed a comparable staining result (Figure 1(b)).

Periodic acid-Schiff (PAS) is a staining method used to detect polysaccharides in tissues, and the reagents in this stain have an affinity for the brush border of the mammalian proximal tubule [35]. Adult zebrafish kidney paraffin sections were stained with PAS, which revealed that their nephrons possessed numerous characteristics conserved with the murine kidney (Figure 1(c)). Namely, the zebrafish proximal tubules were discernible as their brush borders stained a very deep shade of magenta and the cell cytoplasm was dark pink (Figure 1(c)). The basement membranes of the glomerular capillary loops and tubular epithelium were also stained with a similar deep shade of magenta, while epithelial cells of the distal tubule were stained pale pink in color (Figure 1(c)).

Methenamine silver stains are able to detect proteins and have been documented as a marker for basement membranes in mammals [36, 37]. Applying this stain to tissue paraffin sections of the zebrafish kidney, the basement membranes of tubules and glomeruli were visualized by a dark brown hue, and the brush borders of the proximal tubules were also stained dark brown (Figure 1(d)). In addition, the silver stain revealed the presence of hyaline droplets in the proximal tubules, which has also been documented in mammals [38].

### 2.2. Tubule and Duct Compartments Can Be Distinguished Further with Labeling Methods Using Lectins, Myosin VI Expression, and Alkaline Phosphatase Reactivity with ELF-97

Lectins are sugar-binding proteins of nonimmune origin that are expressed throughout nature [39]. Specifically in the kidney organ,* Lotus tetragonolobus* lectin (LTL) marks the proximal tubules, and the targets of* Dolichos biflorus* agglutinin (DBA) include the distal tubules and collecting ducts [39]. Previous studies requiring the ability to distinguish proximal versus distal tubules and quantify such structures in mice have utilized LTL and DBA staining with great success [39–42]. Similarly, for the identification of tubular segments in a medaka fish model of polycystic kidney disease, LTL was used as a proximal tubule marker, and DBA was used as a distal tubule marker [43]. Tissue cryosections of zebrafish adult kidneys were subjected to staining with LTL and DBA (Figures 1(e)–1(i)), as well as whole mount staining (data not shown). The binding specificity of the lectins was conserved in zebrafish, with LTL and DBA labeling distinct tubules (Figures 1(e) and 1(f), resp.), and these labels were mutually exclusive both in cryosection (Figure 1(h)) and in whole mount preparations (data not shown) [32]. Interestingly, the major collecting ducts in the zebrafish kidney, which are a pair of large drainage ducts that extend the entire length of the organ [28], were distinguished via colabeling of LTL and an antibody to detect myosin VI (Figure 1(g)). These were uniquely identified as each zebrafish kidney contained only two such structures, one on each symmetric side of the organ (data not shown).

As proximal tubules possess a brush border, a fluorescence-based method known as ELF- (enzyme labeled fluorescence-) 97 was used to determine if this activity could be localized in adult zebrafish kidneys. The brush borders of epithelial cells in the intestine are known to express high levels of endogenous alkaline phosphatase activity [44]. One previous study reported ELF-97 reactivity in the adult zebrafish kidney, suggesting that ELF-97 staining may be a viable way to label proximal tubule cells [45]. Tissue cryosections of adult zebrafish kidneys stained with the ELF-97 phosphatase were counterstained with the distal tubule marker DBA. The ELF-97 signals were localized to the proximal tubules, which have very prominent brush borders of microvilli that project into the tubule lumens (Figure 1(i), data not shown) [32]. In contrast, tubules that were identified by DBA completely lacked any ELF-97 precipitate (Figure 1(i), data not shown), and these observations were confirmed in whole mount kidney preparations as well (data not shown) [32]. These observations are in agreement with the knowledge that vertebrate nephron distal segments do not possess a brush border and would therefore not be stained with ELF-97 [4, 46].

### 2.3. Histological Characterization of Gentamicin Injury Time Course

Next, we utilized these various histological tools to analyze the phenotype of zebrafish nephrons following AKI. An intraperitoneal injection of gentamicin was administered to induce AKI in adult zebrafish, based on a previously established dosage [28, 29, 47]. The injected fish were sacrificed at several time points and their kidneys fixed, sectioned, and then stained with H&E (Figure 2(a)). At 1 dpi, sufficient nephron damage was induced that resulted in a denuded basement membrane and an immense amount of intraluminal cellular debris. The proximal tubular epithelium had become vacuolated and massive disorganization was evident. Cellular disruption was still apparent at 3 dpi, but within the tubular spaces, cells with a mesenchymal structure were congregated. At 5 dpi, cells within the tubules were more organized, appearing as a visually intact single layer of cells with a hint of a luminal opening. In addition, a small number of basophilic, dark purple cellular clusters had emerged, which is a trait of neonephrogenesis in fish [6]. By 7 dpi, numerous basophilic cellular aggregates had formed, some containing a lumen, and a majority of cellular debris was cleared. Tubular lumens continued to form in the aggregates at 10 dpi and lumens that were evident at 7 dpi had widened. In addition, tubule cells displayed shades of pink similar to that of proximal and distal tubules. By 14 dpi, many of the aggregates possessed obvious brush borders, indicative of a proximal segment, and the kidney tissue overall was analogous to that of an uninjured adult fish. Kidney tissue staining at 21 dpi revealed a similar wild-type appearance with nonexistent cellular aggregates.

A second histological analysis was completed to stain zebrafish kidneys with PAS (Figure 2(b)). PAS was utilized because the stain emphasizes the brush borders of the proximal tubule more effectively than H&E, and thus we hypothesized that this staining method could provide additional insight into the establishment of epithelial character in regenerated proximal tubules. At 1 dpi, the appearance of PAS-positive intraluminal cellular debris was readily observed in distal tubules, as observed in H&E stained samples. Interestingly, hyaline casts have previously been documented as being PAS-positive in the injured kidney of other vertebrate species [48]. Thus, this suggests that the luminal cellular debris was the result of cell death and subsequent sloughing within the renal tubules in areas located upstream of the distal segments, presumably localized to the proximal regions. Kidney tissue at 3, 5, and 7 dpi corresponded closely to that of previously described tissue in the H&E time course. Interestingly, dark magenta linings were noted in many tubules that had only subtle lumens, suggesting they were putative newly regenerated proximal tubules. At 10 dpi, numerous aggregates that have formed possessed a PAS-positive brush border, and by 14 dpi, the kidney tissue was indistinguishable from wild-type tissue. Again, 21 dpi tissue staining revealed an absence of cellular aggregates, suggesting that the regeneration process of neonephrogenesis had been completed. Interestingly, the location of basophilic cellular aggregates that appeared throughout both histological time courses was closely juxtaposed to a nephron tubule (Figure 2). Particularly, at later time points (e.g., 7–10 dpi), each aggregate was associated with a plumbing event into a preexisting proximal tubule based on PAS reactivity, though these events were detectable as early as the 5 dpi time point as well (Figure 2, data not shown). Further, the aggregates themselves displayed the characteristic of PAS reactivity, with dark magenta apical staining (Figure 2, data not shown). This staining character of the basophilic aggregates suggests the largely intriguing notion that new nephrons possess a proximal nature.

### 2.4. *slc20a1a* Expression Time Course in the Injured Kidney

The solute transporter* slc20a1a*, which marks the proximal convoluted tubule (PCT) segment in the adult nephron (Figure 1(a)) as well as the embryo, has been used as a marker of proximal tubule segments after nephrotoxin injury in the zebrafish [29]. To correlate spatiotemporal alterations of this gene with our histology time courses, gentamicin-injected zebrafish kidneys were examined using* in situ* hybridization with* slc20a1a* (Figure 3). At 1 dpi, the gentamicin-induced nephron damage was so catastrophic that* slc20a1a* transcript expression was completely abrogated (Figure 3(a)), consistent with prior observations [29]. The appearance of a small number of* slc20a1a+* cellular aggregates at 3 dpi (0.58 ± 0.28) was followed by substantial increases in this number at 5 dpi (66.66 ± 3.9) and 7 dpi (74.5 ± 9.4) (Figures 3(a) and 3(b)). Small coiled structures first appeared at 5 dpi (2.3 ± 0.55) and were numerous throughout the kidney by 7 dpi (30.6 ± 6.5) (Figures 3(a) and 3(b)). A high number of aggregates also persisted between 7 and 10 dpi; however, the quantity declined as more coils appeared (Figure 3(a), data not shown). Further, the emergence of segment structures, which closely phenocopied uninjured PCT segments, appeared at 7 dpi, with an incidence of 7.9 ± 2.6 structures per kidney (Figures 3(a) and 3(b)). At 14 dpi,* slc20a1a* expression was analogous to that of wild-type adult kidneys, with the majority of structures stained resembling segments (Figure 3(a)). To further analyze these observations, we performed ANOVA statistical analyses to compare the number of aggregates, coils, and segments, which revealed that there was a significant increase in aggregates between 3 and 7 dpi, accompanied by the emergence of coils and segments at 5 and 7 dpi, which was also significant (Figure 3(c)). This suggests that throughout the time period following nephrotoxicant injury, new nephrons first appear as small cellular aggregates that eventually coil and elongate into healthy, normal functioning nephrons, in keeping with the qualitative observations of basophilic aggregates and coils in histological data as well as previous observations of nephron formation [28, 29]. The populace of coils and segments likely also includes the population of existing nephrons that regenerate the damaged proximal tubule epithelium, most likely at time points from 5 dpi onward. However, additional studies are needed to distinguish new nephrons that express* slc20a1a* from existing nephrons that show tubular regeneration.

### 2.5. Cellular Death and Proliferation during Kidney Regeneration

The TUNEL method is a useful and specific label for nuclear DNA fragmentation [49], which is a signature of cellular apoptosis. Previous AKI studies with gentamicin in zebrafish have not examined the timing and location of nephron tubule cell death, though this agent is well known to destroy renal proximal tubule cells. To confirm this directly and to assess whether cell death occurred in other tubule segments, we implemented a combination of TUNEL and LTL labeling to localize when and where cell death occurred in proximal tubules compared to other segments following gentamicin exposure. While uninjured kidneys showed very low levels of TUNEL-positive cells (0.95% ± 0.43%), TUNEL reactivity escalated dramatically in the nuclei of tubular cells, specifically within LTL-positive proximal tubules at 1 and 3 dpi (Figures 4(a) and 4(b)). At 1 dpi, 28.78% ± 1.12% of cells in LTL-positive proximal tubules were TUNEL-positive, which climbed to an incidence of 44.11% ± 6.03% of proximal tubule cells at 3 dpi (Figures 4(a) and 4(b)). By 5 dpi, only 7.82% ± 1.05% of proximal tubule cells were TUNEL-positive (Figures 4(a) and 4(b)). By 7 and 10 days after injection, the level of TUNEL-positive cells in proximal tubules decreased even further, returning to approximately basal levels that had been established in kidneys that were untreated (Figures 4(a) and 4(b)). The rapid elevation in TUNEL reactivity from 1 to 3 dpi followed by rapid decline over 3 to 10 dpi was statistically significant (Figure 4(b)). Renal tubules that were LTL-negative were not found to be TUNEL-positive (data not shown). Similar trends were observed in independent studies in which TUNEL staining was performed in conjunction with ELF-97 to label the proximal tubule (data not shown). Overall, these data show that cell death was occurring largely in the proximal tubule and that it transpires in a wave that peaks at approximately 3 dpi.

Next, we evaluated the dynamics of cell proliferation following gentamicin exposure using PCNA labeling (Figure 5). PCNA is found in varying concentrations within the cell during the cell cycle and is in maximum quantities during the S phase [50]. As with the cell death analysis, the use of LTL labeling in conjunction with the use of an antibody to detect PCNA allowed for examination of cell proliferation in proximal nephron tubules compared to the rest of the renal tubules and ducts. At basal levels in untreated kidneys, 0.97% ± 0.21% of cells were positive in the LTL-positive proximal tubules (Figures 5(a) and 5(b)). At 1 dpi, no cells were found to express PCNA (Figures 5(a) and 5(b)). However, by 3 dpi, 28.6% ± 1.69% of LTL-positive tubular cells were positive for PCNA (Figures 5(a) and 5(b)). The percentage of PCNA positive cells in proximal tubules peaked at 5 days after injury, reaching 60.9% ± 2.16% of all proximal tubule cells. This incidence declined to 24.1% ± 0.51% at 7 dpi and then further to 1.74% ± 0.39% by 10 dpi. PCNA staining was also completed in combination with the proximal tubule marker ELF-97, which showed similar dynamics in the regenerating nephron epithelium, with the proliferation at 3, 5, and 7 dpi (data not shown).

Interestingly, high levels of PCNA were observed in neonephrogenic kidney structures during this time course as well (Figure 5(c)). Beginning at 3 dpi, when aggregates are forming, intense PCNA expression was found in the entire structure colocalizing with DAPI-stained nuclei (Figure 5(c)). By 5 and 7 dpi, when aggregates have formed lumens and transform into coil-like neonephrons, high levels of PCNA were still present (Figure 5(c)). At 10 dpi, PCNA continued to be abundant in these neonephrogenic structures that now had a brush border that stained positive for LTL (Figure 5(c)). Based on these observations, it appears as if the intensely stained PCNA-positive neonephron structures have become proximal tubules. However, genetic fate mapping is needed to definitively track the progression of these structures and label them as having a proximal fate.

As another measure to evaluate cell proliferation, we performed BrdU pulse-chase experiments in zebrafish following gentamicin-induced AKI (Figure 6). For this examination, an intraperitoneal injection of BrdU was administered to uninjured zebrafish and gentamicin-injected zebrafish 24 hours prior to each time point for analysis over a 5-day course, and kidneys were then analyzed by immunofluorescence to detect BrdU-positive cells in proximal tubules labeled with LTL (Figure 6). In uninjured kidneys, we found that 1.25% ± 0.32% of proximal tubule cells had incorporated the BrdU label (Figures 6(a) and 6(b)). After gentamicin injection, at 1, 2, and 3 dpi, there was a low incidence of BrdU incorporation (0.44% ± 0.19%; 0.42% ± 0.18%; 0.83% ± 0.37%, resp.) (Figures 6(a) and 6(b)). At 4 and 5 dpi, however, the incidence of BrdU-positive cells in LTL-stained proximal tubules increased to 5.09% ± 1.16% and 7.42% ± 1.73%, respectively (Figures 6(a) and 6(b)). While ANOVA statistical analysis failed to show that the increase in BrdU incorporation was significant, the overall trend of elevated BrdU incorporation over time in the regenerating proximal tubules is consistent with the observations of PCNA incorporation over time (Figure 5). Taken together, these data show that proliferation in regenerating proximal tubules occurs within the first week following injury.

### 2.6. Restoration of Additional Proximal Tubule Structural Characteristics and Absorptive Functions following Gentamicin-Induced AKI

To further visualize the regeneration of proximal tubule structures following gentamicin-induced injury and to assess the restoration of proximal tubule physiological function, we utilized alkaline phosphatase staining and dextran uptake assays, respectively [32]. In whole mount kidney preparations, these assays enable a three-dimensional assessment of nephrons throughout the organ (Figure 7) [32]. Labeling of alkaline phosphatase activity with ELF-97 specifically enables the visualization of the entire proximal tubule, both the PCT and PST segments, which are connected and have distinguishing diameters, with the PCT being distinctively wide compared to the narrow diameter of the attached PST (Figure 7(a)) [32]. At 1 and 3 dpi, alkaline phosphatase reactivity was diminished and dispersed throughout the kidney, with few PCT structures evident (Figure 7(a)). At 5 dpi and 7 dpi, tubules were more distinctly labeled with alkaline phosphatase, but wide PCT-like tubules were rarely observed (Figure 7(a)). By 14 dpi, there was a clear restoration of PCT-PST structures labeled with alkaline phosphatase reactivity (Figure 7(a)).

In parallel, we examined renal uptake of fluorescently labeled dextran moieties, an assay that determines whether the PCT epithelial cells are capable of endocytosis [32]. Uninjured kidney tubules evinced PCT-specific uptake of fluoro-ruby dextran or fluorescein dextran, while this property was abrogated following gentamicin injury at 1 and 3 dpi (Figure 7(b); data not shown). Between 5 dpi and 19 dpi, PCT uptake of fluoro-ruby or fluorescein dextran was sporadic, with labeling detected in only a few nephron tubules, and not until the 21 dpi time point was PCT uptake consistent across nephrons of the entire organ (Figure 7(b), data not shown). This suggests that regeneration of PCT functionality requires up to three weeks, even though at two weeks the nephron tubules appear structurally intact by alkaline phosphatase labeling (Figure 7(a)) and other histological methods (Figure 2).

### 2.7. Pax2 Expression Marks Regenerating Tubular Epithelial Cells and Neonephrogenic Structures

The process of nephrogenesis is controlled by specific genes that can either enhance or inhibit cell survival and direct subsequent proliferation and differentiation events [51, 52]. One such gene is the* Pax2* transcription factor. During development when renal cells undergo a mesenchymal to epithelial transition into condensed cellular aggregates and differentiate into nephron tubules, they express an increased level of Pax2; later in nephrogenesis, the transcription factor is downregulated [53–55]. In order to restore organ or tissue function in adult animals after undergoing physical damage or injury, it has been suggested that the regeneration process may recapitulate specific developmental processes [56, 57]. In keeping with this notion, previous research has demonstrated that Pax2 is reexpressed in nephron tubular cells following AKI in the adult mouse [58] as well as in zebrafish embryonic nephrons subsequent to gentamicin-induced AKI [59].

In order to test the hypothesis that developmental genes are reexpressed during regenerative events and explore whether zebrafish adult nephrons similarly show tubular Pax2 expression following AKI, an antibody to Pax2 was used for immunolabeling during the adult zebrafish injury and repair time course (Figure 8). Analysis of Pax2 expression in the injured kidney revealed that this developmental transcription factor was expressed in the epithelial tubules at a low level in the uninjured nephron tubule and was more strongly expressed in tubular cells at multiple time points over a two-week time span after injury (Figure 8(a)). Notably, a higher expression level of Pax2 was present between 5 and 7 dpi (Figure 8(a)). A low level was still detectable in the repaired tubules by 14 dpi, comparable to the untreated kidney (Figure 8).

Further, similar to PCNA expression patterns in neonephrogenic structures, Pax2 was expressed at high levels in the cells of coiled bodies and other neonephrogenesis structures at 5 and 10 dpi, respectively (Figure 8(b)). The tubules containing Pax2-positive cells were distinguished from the neonephrogenic structures based on lumen diameter. Tubules that were undergoing repair possessed a larger lumen, representative of a tubule that was previously established and functioning in the kidney. In contrast, lumens of the neonephrons were very small and appeared to expand in time beyond that which is documented in this time course. Taken together, these data demonstrate that expression of Pax2 accompanies regeneration of the proximal tubule epithelium as well as neonephrogenesis in the zebrafish kidney.

## 3. Discussion 

To date, three modes of kidney regeneration have been characterized following exposure to nephrotoxins or mechanical injuries: (1) tubular epithelium regeneration, in which existing nephrons are repopulated after cells have been destroyed, (2) compensatory renal hypertrophy, in which remaining kidney structures enlarge, typically observed following unilateral nephrectomy, and (3) nephron neogenesis from renal mesenchymal progenitor/stem cells [60]. Vertebrate species vary with regard to whether they can perform several or all three of these feats [6, 60]. For example, humans and other mammals are incapable of developing new nephrons following either gestation or the neonatal period, a feature known to be associated with renal stem cell exhaustion during metanephros ontogeny [52, 61–67]. In contrast, fish and amphibians have versatile regenerative traits throughout juvenile stages as well as adulthood that include the formation of entirely new nephrons [10]. While various fish species, including goldfish, medaka, skate, trout, tilapia, and toadfish, can undergo kidney regeneration [33, 68–71], the zebrafish provides an advantage to discover the pathways and signaling events involved in kidney regeneration due to their genetic tractability. Before undertaking traditional genetic or chemical screens using zebrafish to identify the cast of components in renal regeneration, however, it is vital to have a thorough understanding of the progression of tissue changes that transpires following toxicant exposure.

**Figure 9:**
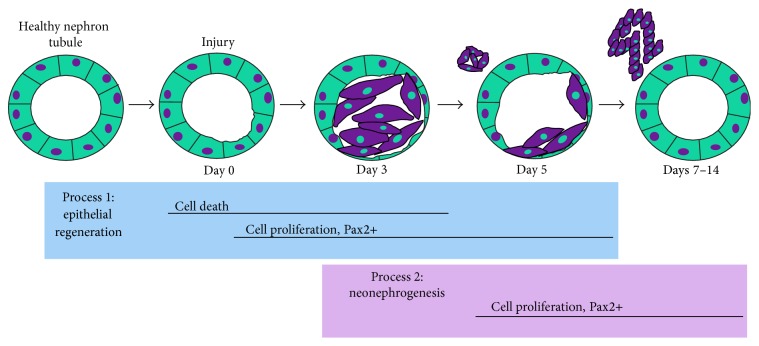
Summary of major cellular events in zebrafish kidney regeneration. After injury to proximal tubules of nephrons within the adult zebrafish kidney, successive and overlapping waves of cell death and cell proliferation occur. Cell proliferation is accompanied by Pax2 expression. Neonephrogenesis entails abundant Pax2 expression and cell proliferation in neonephron structures during the regeneration process.

Here, we have further characterized the spatiotemporal sequence of cellular and gene expression changes associated with regeneration of the zebrafish nephron tubular epithelium and also annotated a number of features associated with neonephrogenesis. In sum, our work has revealed that the injured nephron tubule epithelium is regenerated within one week of damage, involving partially overlapping waves of cell death and proliferation that is accompanied by Pax2 expression (Figure 9). Functionality of the regenerated nephrons is subsequently restored between 2 and 3 weeks following damage. In agreement with prior studies, we found that neonephrogenesis commences by approximately 5 dpi, with nephron clusters forming new nephrons over the subsequent week, and show for the first time that the new nephrons possess the proximal tubule feature of PAS reactivity (Figure 9). While this suggests that new nephrons have proximal character, genetic fate mapping studies are needed to ascertain what functional segment(s) the new nephrons come to possess. Given the highly branched nature of the nephron arrangements in the zebrafish mesonephros, it is an attractive hypothesis that new nephrons commonly plumb into preexisting proximal segments, thus adding to the filtration and bulk reabsorption functions of the kidney while utilizing the existing distal and collecting duct systems for fine-tuning of salt balance in the urinary stream.

### 3.1. Toolkit for Cellular and Molecular Renal Studies in Zebrafish

These studies provide a new and important descriptive atlas of the cellular changes that transpire during zebrafish adult kidney regeneration. Moreover, in the pursuit of these studies we have refined a number of histological methods for their application in the adult zebrafish kidney. Together, this set of information and methodologies provide a resource for further studies in this promising regeneration model. Three histological stains that have proven to be valuable include H&E stain, PAS stain, and silver stain. The H&E stain distinguishes the proximal tubules from the distal tubules based essentially on the presence of a brush border (a marked feature of proximal tubules). The reagents in the PAS stain have a high affinity for the brush borders of proximal tubules and allow a more effective characterization of the varying structures within the kidney tissue. The silver stain also stains the brush borders a discernable dark brown color, allowing distinction from distal tubules. Notably, the silver stain also labels hyaline droplets formed from protein reabsorption, which are located specifically in the proximal tubule. The use of lectin stains to distinguish tubule compartments based on sugar-binding proteins also plays a vital role in this novel toolkit. LTL is a robust proximal tubule marker, labeling the brush borders of the tubule; DBA is a marker for the distal tubules. Finally, the ELF-97 staining method, which detects high levels of endogenous alkaline phosphatase activity in brush borders, is a consistent technique to differentiate the proximal tubules from the distal tubules. A major limitation of working with these and other markers noted above involves incompatibilities with kidney tissue fixation requirements. We have found the most success in zebrafish renal histology procedures by fixing the organ in two different ways: fixation in paraformaldehyde (with or without antigen retrieval prior to immunolabeling) or ethanolic formaldehyde. These methodologies should prove to be useful to further study renal regeneration in the adult kidney, as well as established and emerging models of embryonic nephron injury and regeneration [22, 59, 72–75]. However, not all markers work with both fixatives in combination with immunofluorescent antibodies, and a current limitation in the zebrafish system is the paucity of commercially available antibodies. Future work will mostly likely benefit by examinations of gene expression by additional* in situ* hybridization studies in whole mount or sections [32]. These methods enable the spatial and temporal localization of gene transcripts, which is feasible for any gene(s) of interest because the zebrafish genome has been sequenced and because appropriate reagents are commercially available for gene expression studies.

### 3.2. Stem Cells and Their Roles in Zebrafish Adult Kidney Regeneration

Studies in various mammalian species have demonstrated that intratubular proliferation occurs in the healthy nephron tubule [76–78]. Similarly in the present work, we have documented a low level of proliferation in the uninjured zebrafish proximal tubule based on PCNA reactivity and BrdU incorporation in LTL+ nephron cells. Fate mapping studies in the mouse have clearly demonstrated that intratubular nephron populations replenish the injured nephron [79, 80]. However, the nature of these regenerating cells in the damaged mammalian kidney tubules remains a topic of intense debate. At present, two hypotheses exist that describe the cellular attributes of this intratubular cell source. One scenario involves the dedifferentiation of epithelial cells that will migrate and proliferate to repair injured tubules. The second scenario posits that stem/progenitor cells located within the tubule undergo division and amplification in response to tissue damage. There is experimental evidence among mammalian models that supports both models—fate mapping studies in the mouse support dedifferentiation [8], while there is also opposing evidence from human kidney research that unique subpopulations of renal cells with features suggestive of stem cell character are located among the nephron epithelial cells, and that these cells fuel tubular regeneration [9]. Whether there are differences across mammalian species or whether additional studies will eventually reconcile these conflicting data remains a fascinating area of current nephrology research.

As such, an important aspect of future renal regeneration research with regard to the zebrafish model will be to clarify the origin of reparative epithelia in existing nephrons through transgenic genetic fate mapping. Such lineage analysis will be vital for evaluating the origins of the cells in repaired tubules. Further, since renal cells can be isolated by flow cytometry and then operationally tested through transplantation procedures [81],* in vivo* experiments to test the replicative and differentiation potential of putative intratubular stem/progenitor cells in zebrafish, if they are identified, are likely to become feasible.

### 3.3. Complexities and Benefits in Comparing Zebrafish and Mammals

Ultimately, the degree to which the zebrafish kidney is “unique” from higher vertebrates, including mammals, is an important biological question to understand. For example, the zebrafish adult kidney stroma is the site of hematopoiesis—thus the microenvironment in the zebrafish kidney is arguably quite distinct from mammalian counterparts in which blood production ensues elsewhere. Research that uncovers these or other differences and how they impact renal regeneration capacities may provide vital insights into methods that could be used to stimulate similar reparative responses to treat kidney injury and disease or assist in designing reprogramming strategies [82–84]. The data presented here provide a valuable foundation for researchers that aim to embark on such genetic and cellular studies to ascertain the identities of the molecules and signaling pathways that activate and regulate renal regeneration in zebrafish.

## 4. Experimental Procedures 

### 4.1. Zebrafish Strain and Maintenance

Adult zebrafish of the Tübingen wild-type strain were raised and maintained at 28.5°C on a 14-hour light: 10-hour dark cycle at an average luminance of 200 lux [85] in the Center for Zebrafish Research at the Notre Dame Freimann Life Science Center. All protocols were approved by the IACUC of the University of Notre Dame, animal protocols 13-021 and 16-025.

### 4.2. Gentamicin Injections and Kidney Dissections

For gentamicin injections, zebrafish were anesthetized with a diluted working solution of 0.02% Tricaine, made using a 0.2% Tricaine pH 7.0 stock for approximately 2-3 minutes and transferred to an injection mold. Fish received an intraperitoneal injection of 2.5 mg/mL gentamicin and were returned to a clean system tank. At various time points, adults were euthanized with an overdose of Tricaine and fixed with either 4% paraformaldehyde/1X PBS/0.1% dimethyl sulfoxide (DMSO) or 9 : 1 ethanolic formaldehyde (100% ethanol : 37% formaldehyde). The kidneys of adult fish were dissected as previously described [27, 32]. Briefly, fish were euthanized with 0.2% Tricaine pH 7.0 for approximately 5 minutes. Dissection needles were used to pin open the body walls by attaching them to a dissection tray that contained 4% paraformaldehyde/1X PBS/0.1% DMSO. The samples were fixed overnight at 4°C. The following day, the fixative was removed from the tray and fine forceps were used to detach the kidney from the dorsal wall.

### 4.3. Whole Mount* In Situ* Hybridization

Whole mount* in situ* hybridization (WISH) on adult kidneys was performed as previously described [27, 32]. Briefly, kidneys were fixed in 4% paraformaldehyde/1X PBS/0.1% DMSO and pigmentation in the organ was removed by hydrogen peroxide treatment. Kidneys underwent permeabilization and hybridization steps in a humidified chamber at 70°C overnight. Samples were then incubated in blocking buffer at room temperature (10% bovine serum albumin and 5% fetal calf serum) and following extensive washes, digoxigenin-labeled probes were detected with alkaline phosphatase conjugated to an antidigoxigenin antibody. NBT/BCIP (Sigma-Aldrich) served as the enzymatic substrate for the purple color reaction. Color reactions were stopped with 4% paraformaldehyde/1X PBS.

### 4.4. Tissue Cryosections

As described previously [32], adult fish were fixed in either 4% paraformaldehyde/1X PBS/0.1% DMSO or 9 : 1 ethanolic : formaldehyde overnight at 4°C, and the kidneys were dissected out the next day. Samples were washed with 5% sucrose/1X PBS, cryoprotected in 30% sucrose/1X PBS overnight at 4°C and then subsequently washed in 1 : 1 tissue freezing medium (TFM, Triangle Biomedical Sciences): 30% sucrose/1X PBS overnight at 4°C. The following day, samples were embedded in 100% TFM. Serial sections of approximately 12–14 *μ*m thickness were transversely cut through the entire kidney. Frozen cryosections were mounted onto glass microscope slides (TruBond 380 Microscope Slides, Tru Scientific) and allowed to air-dry for 1 hour at 50°C. Slides were stored at −80°C until use.

### 4.5. Histology Analysis

For all histological stains, adult fish were euthanized at various time points by an overdose of Tricaine and fixed overnight in 4% paraformaldehyde/1X PBS/0.1% DMSO. Kidneys were dissected out, washed in 70% ethanol at 4°C, and then were paraffin-embedded and serially sectioned on a microtome. After slides were deparaffinized and rehydrated, sections were stained with hematoxylin and eosin, periodic acid-Schiff, or methenamine silver (Notre Dame Integrated Imaging Facility–Histology Core). Mouse kidney sections (a generous gift from the Notre Dame Histology Core) were treated with the same histological protocol.

### 4.6. BrdU Incorporation

Cell proliferation was assayed through BrdU incorporation. Adult zebrafish were anesthetized in 0.02% Tricaine pH 7.0 and intraperitoneally injected with 5 mM BrdU (Molecular Probes) 24 hours before sacrifice. Cells that incorporated BrdU were visualized by immunofluorescence analysis.

### 4.7. Immunofluorescence

Slides were thawed for 30 minutes at 50°C and then rehydrated in 1X PBS/0.05% Tween-20. Cryosections were incubated at room temperature in blocking solution 1X PBS/0.05% Tween-20/10% fetal calf serum/1% DMSO for 2 hours and then placed at 4°C for overnight primary antibody incubation. Primary antibodies were diluted in block and included mouse anti-Green Fluorescent Protein monoclonal antibody (1 : 500; Sigma-Aldrich), rabbit anti-Pax2 polyclonal antibody (1 : 50; Covance), rabbit anti-myosin VI antibody (1 : 50; Sigma-Aldrich), mouse anti-BrdU (1 : 50; Molecular Probes), and mouse anti-proliferating cell nuclear antigen (PCNA) polyclonal antibody (1 : 1000; Sigma-Aldrich). Following primary antibody incubation, cryosections were washed in 1X PBS/0.05% Tween-20 and incubated in secondary antibody solution for 2 hours at room temperature. Secondary antibodies were diluted 1 : 500 in 1X PBS/0.05% Tween-20 and included Alexa Fluor 488 and 568 goat anti-mouse IgG and 594 goat anti-rabbit IgG (Molecular Probes). Nuclei were labeled with DAPI (Molecular Probes) for 5 minutes. Cryosections were washed with 1X PBS and mounted with Vectashield Mounting Medium (Vector Laboratories). Antigen retrieval was performed by incubating slides between 95°C and 100°C for 40 minutes in preheated 10 mM sodium citrate buffer for Pax2 and PCNA labeling or by incubating cryosections with 2 M HCl at 37°C for 30 minutes for BrdU labeling. Sections were then washed and immunolabeled as described above.

### 4.8. Identification of Sectioned Tubule Segments

Tubular segments of the kidney were identified by utilizing the following markers: fluorescein* Lotus tetragonolobus* lectin (LTL, Vector Laboratories) diluted 1 : 100 in 1X PBS for 2 hours to label the proximal tubule; enzyme labeled fluorescence- (ELF-) 97 (Molecular Probes) diluted 1 : 20 in detection buffer (included in kit) for 1 hour to label the proximal tubule; rhodamine* Dolichos biflorus* agglutinin (DBA, Vector Laboratories) diluted 1 : 100 in 1X PBS for 2 hours to label the distal tubule [32]. If colabeling with an antibody, LTL and/or DBA stains were applied directly after the secondary antibody incubation. DAPI immunolabeling followed LTL and/or DBA as described above. For ELF-97 colabeling [32], substrate solution was applied directly after the secondary antibody solution. The reaction was stopped with wash buffer 1X PBS/25 mM EDTA/5 mM levamisol pH 8.0, incubating the cryosections with fresh buffer 3 times for 15 minutes each, and then visualized in ELF-97 mounting medium.

### 4.9. Whole Mount Kidney Morphology Assays

#### 4.9.1. Dextran Labeling

Whole mount labeling of proximal convoluted tubule segments in the kidney was followed as previously described [32]. In short, adult zebrafish were anesthetized and injected intraperitoneally with 50 mg/mL fluoro-ruby dextran (Invitrogen) and returned to a clean system tank. The next day, the fish were sacrificed and the kidney was dissected out for fluorescent tubule visualization.

#### 4.9.2. ELF-97 Labeling

Whole mount labeling of pan-proximal segments was performed as previously described [32]. Briefly, kidneys were subjected to fixation, dissection, permeabilization, and pigmentation removal. The kidneys were blocked and then incubated in ELF-97 substrate solution for 30 minutes. Once the reaction was stopped, multiple washes were performed and the fluorescent proximal segments were visualized.

#### 4.9.3. LTL and DBA Labeling

Whole mount labeling of pan-distal segments in the kidney was conducted as previously described [32]. In brief, kidneys were fixed, dissected, and permeabilized and pigment was removed. After blocking, kidneys were incubated in the respective staining solution. Once the staining solution was removed with several washes, the fluorescent signal(s) could be visualized.

### 4.10. TUNEL Assay

Apoptotic cells were identified with the TUNEL assay, using the ApoAlert DNA Fragmentation Assay Kit (Clontech Laboratories) and the TACS 2 TdT Replenisher Kit (Trevigen). Adult zebrafish were fixed in 9 : 1 ethanolic formaldehyde and their kidneys were dissected, embedded, and cryosectioned as previously described. Cryosections were thawed at 50°C for 1 hour, permeabilized with 1X PBS for 20 minutes, 0.1% sodium citrate buffer/0.1% Triton X-100 for 2 minutes, and again with 1X PBS for 5 minutes at room temperature. Equilibration buffer was applied directly to the cryosections for 10 minutes, followed by the addition of biotinylated DNTPs and TdT enzyme (both at a concentration of 1 : 50 in equilibration buffer) for 2 hours at 37°C. The labeling reaction was terminated by incubating cryosections in 2X SSC stop buffer for 15 minutes. Positive nuclei were visualized by applying Alexa Fluor 568 Streptavidin diluted in 1X PBS (1 : 200, Molecular Probes) for 1 hour.

### 4.11. Statistical Analysis

Statistical significance among experimental groups was analyzed using the one-way ANOVA followed by Tukey's HSD multiple comparisons test using R version 3.0.3. Data shown are mean ± SEM. Significance was accepted at *P* < 0.05 or greater.

## Key Findings

- A suite of histological stains was characterized to provide tools to identify distinguishing features of zebrafish adult kidney anatomy, including nephron proximal tubule traits.
- Cell death and proliferation in the injured proximal tubule are dynamic and transpire in successive waves of activity the first week following injury, while functional restoration occurs over the subsequent weeks.
- Spatiotemporal immunofluorescence studies revealed that Pax2 is expressed both in the epithelial population in regenerating nephrons and in neonephrogenic clusters that are associated with the production of de novo nephrons after AKI.

## Abbreviations

AKI: Acute kidney injury
AP: Alkaline phosphatase
BrdU: 5-Bromo, 2′deoxyuridine
CD: Collecting duct
dpi: Days after injury
DAPI: 4′,6-Diamidino-2-phenylindole
DBA: * Dolichos biflorus* agglutinin
DE: Distal early
DL: Distal late
DT: Distal tubule
ELF-97: Enzyme labeled fluorescence-97
G: Glomerulus
H&E: Hematoxylin and eosin
LTL: * Lotus tetragonolobus* lectin
N: Neck
PAS: Periodic acid-Schiff
Pax2: Paired box gene 2
PCNA: Proliferating cell nuclear antigen
PCT: Proximal convoluted tubule
PST: Proximal straight tubule
PT: Proximal tubule
T: Tubule
TUNEL: Terminal deoxynucleotidyl transferase dUTP nick end labeling
WISH: Whole mount* in situ* hybridization.
